# Role of the immune system in the peritoneal tumor spread of high grade serous ovarian cancer

**DOI:** 10.18632/oncotarget.11038

**Published:** 2016-08-03

**Authors:** Katharina Auer, Anna Bachmayr-Heyda, Nyamdelger Sukhbaatar, Stefanie Aust, Klaus G. Schmetterer, Samuel M. Meier, Christopher Gerner, Christoph Grimm, Reinhard Horvat, Dietmar Pils

**Affiliations:** ^1^ Department of Obstetrics and Gynecology, Medical University of Vienna and Comprehensive Cancer Center, Vienna, Austria; ^2^ Department for Laboratory Medicine, Medical University of Vienna, Vienna, Austria; ^3^ Department of Analytical Chemistry, University of Vienna, Vienna, Austria; ^4^ Department of Pathology, Medical University of Vienna, Vienna, Austria

**Keywords:** epithelial ovarian cancer, peritoneal tumor spread, flow cytometry, PD-1, next generation sequencing

## Abstract

The immune system plays a critical role in cancer progression and overall survival. Still, it is unclear if differences in the immune response are associated with different patterns of tumor spread apparent in high grade serous ovarian cancer patients and previously described by us. In this study we aimed to assess the role of the immune system in miliary (widespread, millet-sized lesions) and non-miliary (bigger, exophytically growing implants) tumor spread. To achieve this we comprehensively analyzed tumor tissues, blood, and ascites from 41 patients using immunofluorescence, flow cytometry, RNA sequencing, multiplexed immunoassays, and immunohistochemistry. Results showed that inflammation markers were systemically higher in miliary. In contrast, in non-miliary lymphocyte and monocyte/macrophage infiltration into the ascites was higher as well as the levels of PD-1 expression in tumor associated cytotoxic T-lymphocytes and PD-L1 expression in tumor cells. Furthermore, in ascites of miliary patients more epithelial tumor cells were present compared to non-miliary, possibly due to the active down-regulation of anti-tumor responses by B-cells and regulatory T-cells. Summarizing, adaptive immune responses prevailed in patients with non-miliary spread, whereas in patients with miliary spread a higher involvement of the innate immune system was apparent while adaptive responses were counteracted by immune suppressive cells and factors.

## INTRODUCTION

Ovarian cancer is the deadliest gynecological malignancy in the Western world and the sixth leading cause of cancer related deaths among women [1]. Despite efforts to find biomarkers for early diagnosis of epithelial ovarian cancer (EOC), the majority of patients present with already advanced disease at the time of first hospitalization, characterized by a high and widespread tumor load in the peritoneal cavity, often accompanied by malignant ascites. Ascites provides a specialized tumor microenvironment with low oxygen levels that harbors not only tumors cells but also cancer-associated fibroblasts, reactive mesothelial cells from the peritoneal wall, and immune cells in variable proportions [2]. Previous studies have shown that inflammation regulating factors and the presence of various immune cell populations affect the clinical outcome of EOC patients [3]. Most importantly, infiltration of (CD8+) T-cells into ovarian tumor tissue proved to be a positive predictive factor for overall survival [4].

CD8+ cytotoxic T-cells are a major subpopulation of T-cells capable of mediating anti-tumor immune responses in ovarian cancer. The activity of cytotoxic T-cells is regulated by immune-modulatory signals, that can be co-stimulatory (CD80 or CD86 binding to the receptor molecule CD28 on T-cells) or inhibitory (CTLA-4 competing with CD86 and CD80 for CD28). Apart from T-lymphocytes, NK cells are known for their anti-tumor functions. NK cells have different phenotypes, that can be differentiated according to their CD56 and CD16 expression on the cell surface: The CD56^dim^CD16+ subtype is the main type of NK cells found in blood and has cytolytic functions whereas the CD56^bright^CD16- subtype is prevalent in secondary lymphoid organs and possesses only minor cytolytic activity but was shown to secrete different effector molecules (IL-10, IFNγ, TNFα, and GM-CSF) [5].

Tumor cells can achieve the downregulation of immune responses by production of immune suppressive cytokines or a failure to produce costimulatory molecules, leading to tolerance or anergy of T-cells. Additionally, tumor reactive cells can be killed or silenced by tumor cells expressing inhibitory molecules like programmed death-ligand 1 (PD-L1). Lymphocytes, especially T-cells, were shown to upregulate the receptor programmed death-1 (PD-1) after activation. Binding of the ligands PD-L1 or PD-L2, shown to be expressed by certain tumor cells, to the corresponding receptor induces T-cell anergy, unresponsiveness, and apoptosis of T-cells [6] and therefore leads to downregulation of the anti-tumor immune response.

Despite the different histologically diverse epithelial ovarian cancers, even the group of high grade serous ovarian cancer (HGSC) shows a high molecular heterogeneity. Gene expression profiling and subsequent clustering led to the establishment of six molecular subclasses of HGSC by Tothill *et al.* [7] and four of these six subclasses were additionally evaluated by The Cancer Genome Atlas Project (TCGA). The HGSC specific clusters were termed in accordance with their gene expression signatures: C1 (mesenchymal), C2 (immunoreactive), C4 (differentiated), and C5 (proliferative) [8]. Significant differences in survival between these subtypes were only discovered in a subsequent study, updating the clusters with additional prognostic signatures. Comparing the clusters, the immunoreactive (C2) subtype showed the best survival, presumably because it is associated with high numbers of tumor infiltrating lymphocytes [9, 10].

Recently, we proposed a new classification of HGSC on the basis of different types of peritoneal tumor spread [11, 12]. We could show that patients, presenting either without peritoneal tumor implants (in addition to the ovarian tumor mass) or with only few, but bigger (>2 cm) and exophytically growing tumor implants vary from patients presenting with numerous, small (<2 cm) peritoneal lesions in terms of survival, molecular characteristics, and clinical appearance. We developed gene and small RNA expression signatures for tumor spread and proved, that the non-miliary type showed favorable overall survival, independent of typical clinicopathologic factors, whereas miliary tumor cells correlated significantly with an enhanced epithelial status [11, 12]. The next step was to analyze the impact of the microenvironment and immune system on tumor spread. Here we present an integrative analysis of the different microenvironmental factors in ovarian cancer using flow cytometric analyses of lymphocyte populations in ascites and tumor tissues, multicolor immunofluorescence (IF) staining of ascites monocytes, RNA sequencing (RNA-seq) results of CD45-enriched immune cells from tumor tissues and ascites, and analysis of chemokines using multiplexed immunoassays. In addition, a targeted metabolomics approach from cell free ascites and blood revealed differences between both tumor spread types. The comprehensive results allowed us to compare the microenvironment of the two spread types miliary and non-miliary and revealed clear differences about the involvement of the adaptive and the innate immune system in tumor spread.

## RESULTS

### Patients, samples, and experimental design

We were the first to comprehensively analyze the microenvironment of HGSC with respect to tumor spread. Therefore, numerous samples of immune cells and tumor cells from spatially diverse origins (blood (B), ascites (A), tumor tissues from ovarian (P, for “primary”) and peritoneal tumors (M, for “metastasis”)) and cell free supernatants were analyzed. Forty-one patients suffering from HGSC were consecutively included in this study. The majority (90%) presented with advanced disease, *i.e.* FIGO III/IV (Table 1). According to our proposed definition of peritoneal tumor spread [11] 20 patients (48.8%) showed miliary tumor spread and 15 patients (36.6%) showed non-miliary spread. In six patients (14.6%) the tumor spread was indeterminable, either because of very advanced disease with a large tumor burden in the peritoneal cavity or because it was not assessed during surgery. All analyses were performed on this patient cohort in order to achieve a comprehensive analysis of miliary and non-miliary tumor spread in different compartments. Additionally, blood samples from ten healthy females and ascites samples from nine patients with cirrhotic or non-cirrhotic portal hypertension but without malignant background were collected as control for flow cytometric (FACS) analysis. For an overview of immune cell and tumor cell content in ascites, formalin-fixed, paraffin-embedded (FFPE) ascites samples were analyzed using IF. To further analyze the composition of the lymphocyte population, ascites, blood, and tumor cell depleted tumors from the same cohort as above were subjected to multicolor FACS analysis. Non-cellular factors in ascites and blood of these patients were assessed with multiplexed immunoassays and standard laboratory tests for C-reactive protein (CRP), albumin, and low- and high-density lipoproteins (LDLs and HDLs) in order to gain information about inflammatory and other immune-regulatory parameters. Furthermore, transcriptomes of immune cells and tumor cells from a subset of the patient cohort were analyzed with RNA-seq to describe connections and differences in gene expression. Among others, PD-1 and PD-L1 gene expression was analyzed in all available tissues (ascites, blood, and ovarian/peritoneal tumors) and compared to results from IHC staining of primary tumors and peritoneal implants from the same patients. Lastly, to assess metabolic changes accompanying different patterns of tumor spread, levels of selected metabolites were analyzed in serum and ascites samples.

**Table 1:** Patients' characteristics

Characteristic	Number of Patients (%)
**Histology**	
Serous	41 (100)
**TP53 mutation**	
yes	36 (87.8)
no	2 (4.9)
Unknown	3 (7.3)
**Grade**	
2	7 (17.1)
3	32 (78)
unknown	2 (4.9)
**FIGO**	
IB	1 (2.4)
IIA	1 (2.4)
IIC	1 (2.4)
IIIA	1 (2.4)
IIIB	4 (9.8)
IIIC	25 (60.9)
IV	7 (17.1)
Unknown	1 (2.4)
**Lymph nodes**	
Positive	13 (31.7)
Negative	10 (24.4)
Unknown	18 (43.9)
**Spread**	
non-miliary	15 (36.6)
miliary	20 (48.8)
Unknown	6 (14.6)
**Ascites**	
No	8 (19.5)
<500ml	14 (34.1)
>500ml	19 (46.3)

### Analysis of ascites cell populations using IF staining

**Figure 1:**
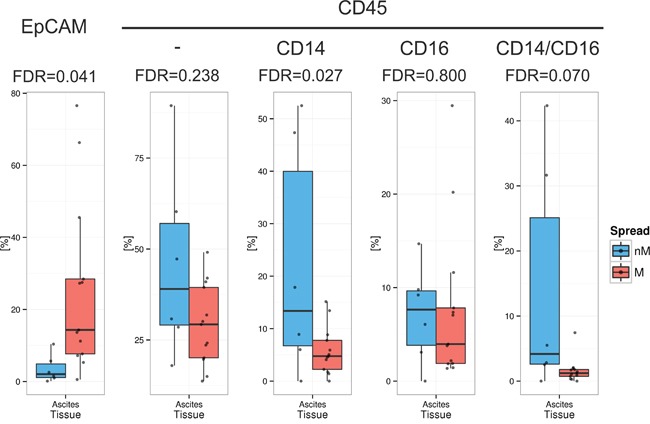
Frequency of cell populations in malignant ascites Quantification of tumor cells and immune cells in ascites (n=19), separated in non-miliary (nM, blue) and miliary (M, red). Boxplots represent the calculated frequencies of the respective cell populations in percent of total cells. Populations from left to right are EpCAM+ tumor cells, CD45+ immune cells, CD45+CD14+ MO/MA, CD45+/CD16+ cells, and CD45+CD14+CD16+ differentiated MO/MA.

To assess immune and tumor cell content and frequency of monocytes and macrophages (MO/MA) in ascites, FFPE ascites cells were stained for the immune cell marker CD45, the tumor cell marker EpCAM, and the MO/MA associated markers CD14 and CD16 using IF. Levels of EpCAM+ tumor cells, CD45+ immune cells, CD45+/CD14+ MO/MA, and CD45+CD14+/CD16+ differentiated MO/MA were quantified by automated cell image analyses and compared between samples from miliary (n=6) and non-miliary (n=13) ascites. Although frequencies of immune cells did not differ significantly between miliary and non-miliary ascites (false discovery rate, FDR=0.238) higher levels of CD14+ MO/MA and differentiated CD14+CD16+ MO/MA could be observed in non-miliary (FDR=0.027 and FDR=0.070, respectively; Figure 1). Levels of CD45+CD16+ cells did not differ significantly between miliary and non-miliary. On the contrary, EpCAM positive tumor cells were enriched in miliary ascites (FDR=0.041; Figure 1). Together, these data indicate a MO/MA rich microenvironment in non-miliary compared to miliary ascites, which in turn showed higher levels of tumor cells.

### Identification and comparison of immune cell populations in blood and ascites using flow cytometry

We determined the composition of the lymphocyte population in HGSC patients by analyzing 26 different sub-populations in ascites (n=26) and blood samples (n=30) using two multicolor flow cytometry panels. Levels of lymphocyte sub-populations were compared between samples from miliary and non-miliary tumor spread and also compared to healthy controls (ascites, n=9 and blood, n=10). Analyzed cell populations included CD3+ lymphocytes, separated (gated) into cytotoxic T-cells (CD8+), T-helper cells (Th, CD4+), regulatory T-cells (Tregs) and natural killer T-cells (NKT, HLA-DR+ and -). CD3- lymphocytes were gated into B-cells (CD19+, naïve and memory), natural killer (NK) cells (CD56+), and five different subsets of NK cells (each HLA-DR+ and -; see Supplementary Table S1).

**Figure 2:**
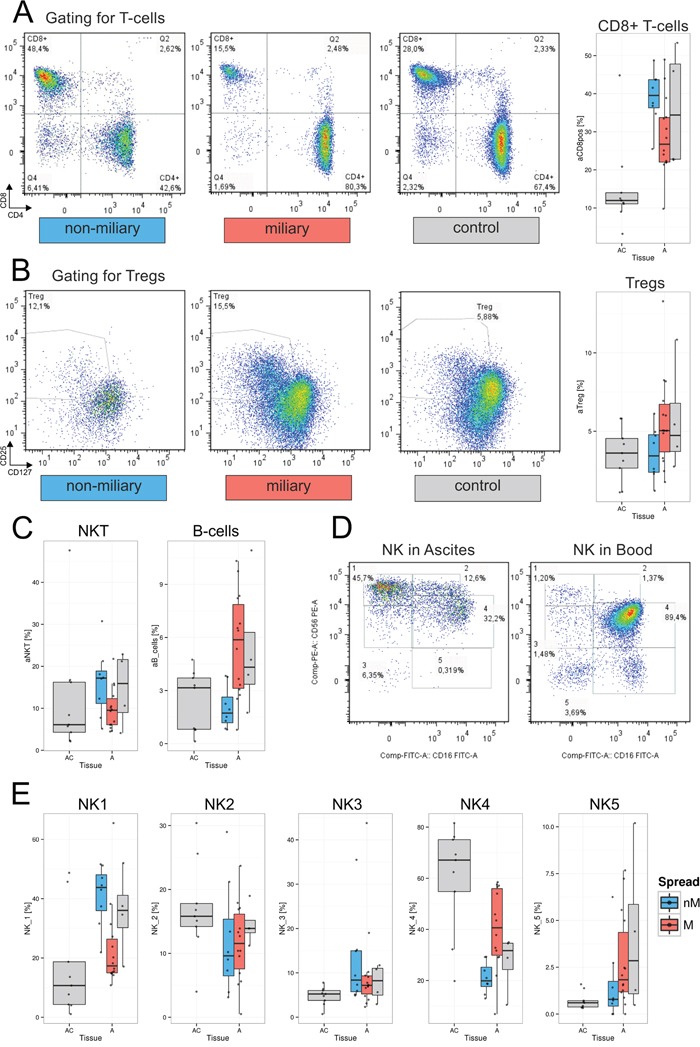
Lymphocyte populations in ascites and blood **A.** Gating of representative samples for each, non-miliary (blue), miliary (red), and control ascites: A lymphocyte gate was set and lymphocytes were gated for live cells and CD3+ T-lymphocytes. The depicted gate shows CD4 versus CD8 expression. CD8+ T-cells are shown here as CD3+CD8+CD4- cells. Right, boxplots indicating the total frequencies of CD8+ cells in ascites in percent of lymphocytes. BC, control blood; B, blood from HGSC patients; AC, ascites control; A, malignant ascites; grey, blood/ascites with indeterminable spread type. **B.** Gating of representative sample for Tregs. The dot plot depicts CD4+ cells discriminated according to CD127 and CD25 positivity. Right, boxplots showing the total frequencies of Tregs in ascites in percent of total lymphocytes. **C.** Boxplots representing the frequencies of NKT-cells (left) and B-cells (right) in percent of total lymphocytes. **D.** Representative gating of NK cell subpopulations in ascites (left) and blood (right). Cells from a lymphocyte gate were gated for CD3- and CD19-negativity. The depicted gate shows CD56 versus CD16 expression. The five different NK cell subtypes are NK1, CD56^bright^CD16-; NK2, CD56^bright^CD16+; NK3, CD56^dim^CD16-; NK4, CD56^dim^CD16+; and CD56-CD16+. **E.** Boxplots representing the frequencies of NK cell subpopulations in frequencies of CD3-CD19- cells.

The frequencies of single lymphocyte sub-populations in blood and ascites samples are described below. The levels of several sub-populations differed statistically significantly (FDR<0.2) in ascites between miliary and non-miliary including CD8+ cells, regulatory T-cells (Tregs), NKT-cells, naïve B-cells, and two subtypes of NK cells (Figure 2), whereas in blood, no significant differences were found.

#### CD8+ cytotoxic cells

Frequencies of CD3+ T-cells were similar in blood and ascites of HGSC patients accounting for a median of 81% (range: 65%-88%) of lymphocytes in blood and 83% (range: 64%-88%) of lymphocytes in ascites. Stratified for tumor spread, patients with non-miliary tumor spread showed a significantly higher frequency of CD8+ cells in the ascites than patients presenting with miliary spread (FDR=0.150). In comparison to control ascites, both, miliary and non-miliary ascites showed significantly elevated frequencies of CD8+ cells (FDR=0.006; Figure 2A). While no significant differences of CD8+ cell frequencies in miliary and non-miliary blood were found, CD8+ cell levels of matched blood and ascites samples from the same patients correlated positively (*r*=0.55, p<0.05; Supplementary Figure S1).

Total CD3+ and CD4+ cell frequencies were not correlated between matched ascites and blood samples, but looking at the whole HGSC patient cohort, CD4+ cells were more frequent in blood than in malignant ascites.

#### Regulatory T-cells

Tregs were found in all samples with the highest frequency in ascites of the miliary spread type. The levels of Tregs in non-miliary ascites were similar to those of control ascites (Figure 2B). In blood, differences between miliary and non-miliary T-cell frequencies were not significant but a trend towards elevated Tregs in miliary was observed, whereas again, the levels in control blood and non-miliary were similar. A moderate, statistically significant positive correlation of blood and ascites Treg levels was found from matched patients' samples (*r*=0.63, p<0.01; Supplementary Figure S1).

#### Natural killer like T-cells

The level of NKT cells was significantly enriched in non-miliary ascites as compared to miliary and control ascites (FDR=0.170; Figure 2C). In blood, NKT levels were similar comparing control and malignant blood and there was a moderate positive correlation in matched ascites and blood samples (*r*=0.53, p<0.05; Supplementary Figure S1).

#### B-cells

Among lymphocytes, higher frequencies of B-cells were present in controls and patients' blood than in ascites (Supplementary Figure S1). In malignant ascites, a distinct trend towards B-cell enrichment in miliary compared to non-miliary was found (FDR=0.275; Figure 2C). Blood levels of B-cells were similar comparing miliary and non-miliary. Interestingly, we could observe a trend towards a low negative correlation between frequencies of B-cells in blood and ascites (*r*=−0.42, p<0.1; Supplementary Figure S1) and comparing B-cell subpopulations, a significantly lower level of CD27+CD19+ memory B-cells was present in blood from miliary compared to non-miliary and also compared to control blood (FDR=0.040; Supplementary Figure S1).

#### Natural killer cells and subpopulations

Two different gating approaches were used to analyze NK cells: Total NK cells were defined as CD3-CD19- cells, positive for the NK-cell marker CD56. Furthermore, five different subpopulations of NK cells were gated as described by Poli *et al.* [13] according to the relative expression of the surface markers CD56 and CD16 (Figure 2D). Total NK cell frequencies were similar in patients' ascites and blood, as well as in control blood, but elevated in non-malignant ascites, used as a control (Supplementary Figure S1). As described before [14], the levels of the two main NK cell populations, CD56^bright^CD16- (NK1) cells and CD56^dim^CD16+ (NK4) cells, differed most between patients' blood and ascites but only the CD56^bright^CD16+ (NK2) cell levels correlated positively between blood and ascites (*r*=0.7, p<0.01). The NK4 cells constituted the most prominent NK-subpopulation in patients' blood, accounting for two thirds of CD3-CD19- cells (median 66.8%; range 20.9%-86.1%). In malignant ascites, this population was substantially smaller accounting for less than one third of CD3-CD19- cells (median of 29.3%; range 6.9%-58.5%). Significant differences in the frequencies of NK cell subsets were observed in malignant ascites. Frequencies of the cytotoxic CD56^dim^CD16+ (NK 4) cells among NK cells were significantly higher in miliary ascites (FDR=0.033), whereas levels of cytokine producing CD56^bright^CD16- (NK1) cells were significantly higher in non-miliary ascites (FDR=0.004; Figure 2E).

### Non-cellular factors in ascites and blood

**Figure 3:**
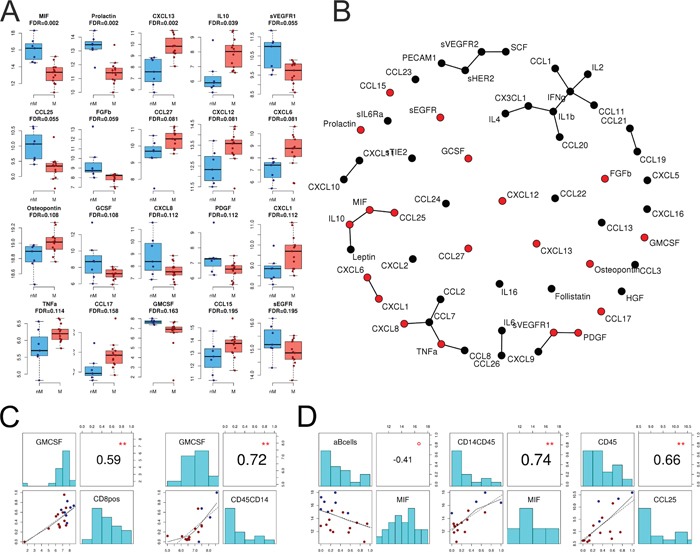
Chemokines and cytokines in ascites **A.** Levels of 20 significantly different chemokines and cytokines between miliary and non-miliary in ascites as assessed by multiplexed immunoassays. Non-miliary (nM), blue; miliary (M), red. **B.** Co-occurrence network (Graphical Gaussian Models) showing all 56 assessed chemokines and cytokines in ascites; statistically significant in red. **C.** Correlations of immunoassay data with FACS and IF results. Left: correlation of GM-CSF in ascites with the frequency of CD8+ cells in ascites (FACS); right: correlation of GM-CSF in ascites with the frequency of CD45+CD14+ MO/MA (IF). **D.** From left to right: Correlation of MIF levels and B-cell frequency in ascites (FACS), correlation of MIF levels and CD14+CD45+ MO/MA (IF) in ascites, and correlation of CCL25 with CD45+ immune cells in ascites (IF). Colors of dots in C and D correspond to tumor spread type: red, miliary; blue, non-miliary. Correlations are Spearman's correlation coefficients and p-values (**p<0.01, *p<0.05, °p<0.1).

A total of 56 human chemokines and cancer biomarkers (soluble receptors, cytokines, chemokines, growth factors, and hormones) were analyzed in samples of human blood serum (n=23) and ascites (n=20) from 28 HGSC patients using multiplexed immunoassays (Supplementary Table S2). In ascites, the level of 20 chemokines and growth factors differed significantly (FDR <0.2) between miliary and non-miliary (Figure 3A). The highest differences were seen in levels of CXCL13 with a 2.23 log_2_-fold enrichment in miliary, and macrophage migration inhibitory factor (MIF), with a 2.84 log_2_-fold enrichment in non-miliary (Figure 3A). In serum, no significant differences between chemokine levels could be detected comparing miliary and non-miliary. Based on correlation coefficients and corrected for global correlation levels (so called Graphical Gaussian Models), networks of associated chemokines were constructed (Figure 3B). One network consisted of leptin, IL-10, MIF, and CCL-25. Levels of all factors, except for leptin, differed significantly between miliary and non-miliary. Leptin, synthesized by white adipose tissue [15], activates B-cells (enriched in miliary ascites) to secrete IL-10 [16] (enriched in miliary ascites). IL-10, on the other hand, inhibits MIF synthesis (low in miliary) and therefore recruitment of MO/MA (down in miliary) [17]. Further, CCL-25 (down in miliary) is associated with leukocyte recruitment (thymocytes, macrophages, dendritic cells (DC), but not peripheral blood mononuclear cell) and the lower levels of CCL-25 in miliary ascites might explain the low abundance of CD8+ T-cells, NKT and MO/MA. Indeed, the frequency of B-cells in ascites correlated negatively with the level of MIF (*r*=−0.41, p<0.1), whereas the MIF levels correlated positively with the frequency of MO/MA in ascites (*r*=0.74, p<0.01), and CCL-25 levels correlated positively with the frequency of leukocytes (*r*=0.66, p<0.01; Figure 3D). Further cytokine networks with spread-type association were CXCL8-CCL7-CCL2 and PDGF-VEGFR1-CXCL9.

As activated CD8+ cells are known to produce granulocyte-macrophage colony-stimulating factor (GM-CSF) [18, 19] GM-CSF levels were compared to the abundance of CD8+ cells. In matched ascites samples, levels of CD8+ cells (as defined by FACS) and GM-CSF correlated positively (*r*=0.59, p<0.01). Also, the ascites levels of CD45+CD14+ cells (IF) and GM-CSF (*r*=0.72, p<0.01) correlated positively. GM-CSF and CD45+CD14+ cells were both significantly enriched in non-miliary ascites compared to miliary (Figure 3C).

A non-linear dimensionality reduction approach (Isomap) using significantly different ascites analytes revealed a separation of samples according to the two spread types along dimension one (Supplementary Figure 2A). Concentrations of MIF, soluble EGFR, leptin, IL-6, CXCL10, and CXCL11 correlated positively between ascites and serum (Supplementary Figure 2B).

In order to gain further information about systemic and local inflammation, levels of CRP were assessed in serum (n=24) and ascites (n=21) of 29 HGSC patients. In ascites, CRP was significantly enriched in the miliary spread type as compared to non-miliary (p=0.025). In serum, CRP was enriched non-significantly in miliary, although a distinct trend was observed (p=0.130). Nevertheless, paired ascites and serum samples (n=16) revealed a strong positive correlation of CRP levels (*r*=0.92, p<0.001). High levels of CRP were previously linked to reduced overall and disease-free survival in ovarian cancer [20], which is in concordance with the reduced overall survival of patients with miliary spread type, as shown by our group [11, 12]. Furthermore, LDL was significantly enriched in ascites from miliary compared to non-miliary (p=0.009) and also correlated positively with matched serum samples (*r*=0.72, p<0.01). Also, levels of albumin (*r*=0.75, p<0.001), cholesterol (*r*=0.74, p<0.01), and HDL (*r*=0.62, p<0.05) correlated significantly between serum and ascites (Supplementary Figure S3). Furthermore, albumin levels were lowered in miliary in both, ascites and serum, albeit not significantly (p=0.2 and p=0.3, respectively; Supplementary Figure 3).

### Identification and comparison of immune cell populations in cancerous tissues using flow cytometry

**Figure 4:**
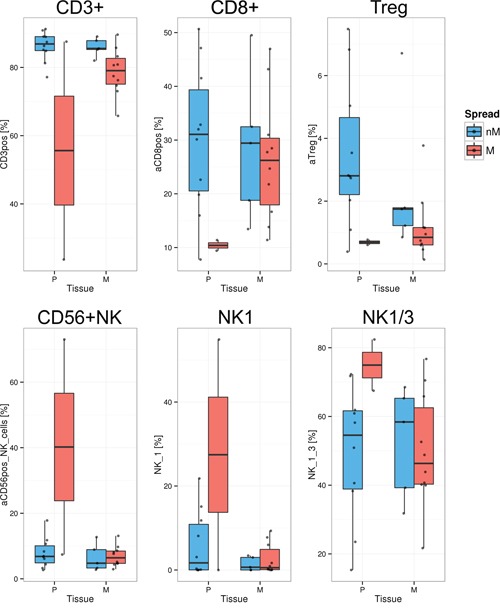
Significantly different lymphocyte populations in tumor tissues (FDR<0.2) Quantification of lymphocyte populations in EpCAM depleted (n=19) single cell solutions from primary tumors (P) and peritoneal implants (M, metastasis) as assessed by FACS, separated in non-miliary (nM, blue) and miliary (M, red). Boxplots represent the calculated frequencies of the respective cell populations in percent of total lymphocytes, except for NK1 and NK1/3 (percent of CD3-CD19- cells). Upper panel: CD3+ T-cells, CD8+ T-cells, and Tregs; Lower panel: CD56+ NK-cells, NK1 cells, and NK1 plus NK3 populations together.

Levels of the same 26 lymphocyte populations as above were analyzed in digested and EpCAM+ cell depleted tumor tissues of ovarian (n=12) and peritoneal (n=15) origin from 20 HGSC patients (Supplementary Table S1). Comparing tissues from miliary (n=12) and non-miliary (n=15) tumor spread, the most obvious difference was the higher frequency of CD3+ cells among lymphocytes in the latter (FDR=0.004; Figure 4). Consequently, also higher levels of CD8+ T-cells were found in non-miliary (FDR=0.114). Furthermore, we found higher frequencies of Tregs in tumor associated lymphocytes in non-miliary, as opposed to ascites, although only small numbers of CD4+ positive cells were observed within tissue samples (*i.e.* median of 7.7% versus median of 48% of lymphocytes in blood, respectively; Supplementary Figure S1). There were no correlations of frequencies of lymphocyte populations from tumor tissues and ascites but a strong, positive correlation of CD8+ cells in tissue and blood (r=0.73, p<0.05), albeit only when analyzing frequencies of CD8+ per CD3+ and not the total frequency of CD8+ cells among lymphocytes (r=0.28, p>0.1; Supplementary Figure S1). In miliary, the frequency of NK cells and especially the NK1 subpopulation was higher compared to non-miliary (NK, FDR=0.008 and NK1, FDR=0.114; Figure 4). Tumor associated and tumor infiltrating NK cells were recently discussed to play a pro-tumorigenic role and display a compromised cytolytic phenotype [21]. The most prevalent NK subtype found in our samples was the CD56^bright^CD16- subtype which possesses only minor cytolytic activity but is able to produce cytokines, a function possibly hijacked by the tumor to produce pro-tumorigenic factors.

### Transcriptome analysis

#### Immune cells

Poly-A positive RNAs from 39 immune cell enriched tissue samples (A, P, and M) from 20 patients were sequenced to a median depth of 21 mio 50 bp paired end-reads (range: 6-30 mio). After filtering, mapping of the reads, and counting into a gene model, 14,657 reliably expressed genes were used for subsequent analyses. Comparing gene expression between miliary (n=11) and non-miliary (n=9) and taking tissue origins into account, 18 genes were shown to be significantly differentially expressed (FDR<0.2), with five down- and 13 upregulated genes in miliary (Supplementary Table S3).

In CD45 enriched immune cells from solid tumor tissues (P/M), only seven significantly differentially expressed genes (FDR<0.2) were found between miliary (n=12) and non-miliary (n=13), three down- and four upregulated. In contrast, immune cells from ascites (9 miliary versus 5 non-miliary samples, respectively) showed 33 significantly differentially expressed genes (FDR <0.2), 17 of them down- and 16 upregulated in miliary (Supplementary Table S3). The majority of these genes was protein coding. Among the most downregulated genes in miliary were FABP4 (FABP4 fatty acid binding protein 4) and OGN (osteoglycin).

Comparing these gene expressions to results from the above described flow cytometric analyses we found that the expression levels of 395 genes were significantly correlated to the elevated frequencies of NKT cells in non-miliary ascites (FDR<0.2). Only 38 of these genes were negatively correlated but 357 genes correlated positively. Subjecting these significantly correlated genes to SPIA pathway analysis yielded following four pathways: Focal adhesion, PI3K-Akt signaling pathway, Rap1 signaling pathway, and ECM-receptor interaction, all activated.

#### Tumor cells

**Figure 5:**
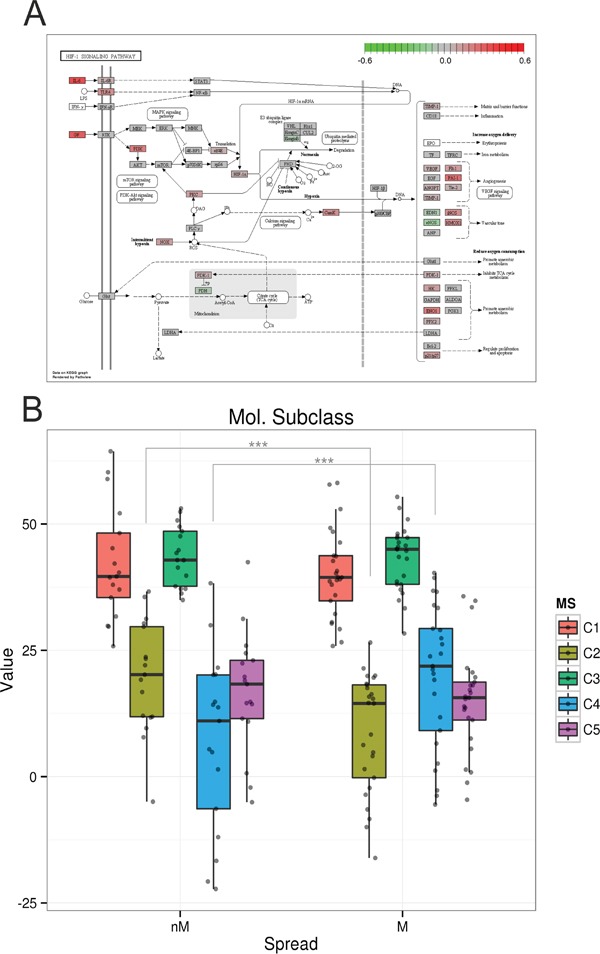
HIF signaling pathway and molecular subclasses [7] **A**. KEGG pathway plot of the HIF signaling pathway. Colors correspond to the correlations of gene expression values associated with the frequency of NKT cells in ascites (red positively and green negatively correlated). **B.** Classification of non-miliary and miliary tumor cells from primary and metastatic tumors according to the molecular subtypes C1-C5. Asterisks mark significantly differentially distributed subtypes between miliary and non-miliary. ***p<0.001.

Comparing the gene expressions of tumor cells [11], with NKT frequencies revealed 128 significantly correlated genes, 14 negatively and 114 positively. SPIA analysis of these data revealed 19 associated pathways, with the PI3K-Akt signaling pathway as the most significant one (Table 2). Among others, the HIF-1 signaling pathway was associated with the frequency of NKT cells (Figure 5B). HIF-1 signaling was described to increase oxygen delivery under hypoxic conditions by induction of angiogenesis [22]. Furthermore, an increased activation of NKT cells via induction of HIF in hypoxic environments was described previously [23].

**Table 2:** SPIA pathway analysis. Pathways associated with the level of NKT cells in ascites. FDR, false discovery rate

Name	FDR	Status
PI3K-Akt signaling pathway	1.52E-06	Activated
Focal adhesion	2.34E-05	Activated
Melanoma	0.001857056	Activated
FoxO signaling pathway	0.010840666	Activated
Rap1 signaling pathway	0.010840666	Activated
ECM-receptor interaction	0.010840666	Activated
Prostate cancer	0.015608191	Activated
Pathways in cancer	0.02876967	Activated
HIF-1 signaling pathway	0.034601726	Activated
Choline metabolism in cancer	0.035880867	Activated
Cytokine-cytokine receptor interaction	0.039910602	Activated
Gap junction	0.040003465	Activated
TGF-beta signaling pathway	0.074810141	Inhibited
HTLV-I infection	0.098892461	Activated
MAPK signaling pathway	0.122081826	Activated
AMPK signaling pathway	0.149755218	Activated
Aldosterone-regulated sodium reabsorption	0.159611663	Activated
Proteoglycans in cancer	0.195712253	Inhibited

Comparing gene expression of tumor-cells, enriched for EpCAM positive cells, with molecular subtypes as defined by Tothill *et al.* [7], we found a significant association of non-miliary tumor cells with the immunoreactive C2 subtype (p<0.001), whereas miliary tumor cells were significantly associated with the differentiated or C4 subtype (p<0.001, Figure 5A). In a similar analysis, also gene signatures for TCGA subclasses [8] and angiogenesis types [24] were shown to be significantly associated with our classification according to tumor spread. Miliary spread was associated with the “high risk” subclass with worse overall survival in TCGA (p=0.002), and non-miliary with the increased angiogenesis subclass (p=0.040; Supplementary Figure S4 and Supplementary Table S4).

#### PD-L1 and PD-1 expression

Programmed death-1 (PD-1) is expressed mainly on activated T cells and involved in suppression of T-cell effector functions [25]. The ligand PD-L1 was shown to be expressed on a variety of cell types and also tumor cells as a strategy to repress T-cell mediated immune responses [6]. The protein PD-L1 is encoded by the gene PDCD1 and PD-1 is encoded by the gene CD274. For better readability, both, genes and protein, are referred to with their protein names.

Comparing results from RNA-seq of CD45 enriched cells with flow cytometry data revealed a positive correlation of the receptor PD-1 with the frequency of CD3+ T-cells among lymphocytes (*r*=0.59, p<0.001). Differentiating between miliary and non-miliary, PD-1 mRNA expression was significantly higher in samples of the non-miliary spread type (p=0.030) and also PD-L1 expression was slightly enriched in non-miliary (p=0.060; Supplementary Figure S5A).

Transcriptome analysis of samples that were enriched for tumor cells (EpCAM) revealed a significantly higher expression of PD-L1 mRNA in non-miliary compared to miliary (p=0.010). Expression of PD-1 was also enriched in non-miliary, albeit not significantly (p=0.060; Supplementary Figure S5B). Comparing FACS data of immune cells with transcriptome data from tumor cells, no significant correlation of PD-1 or PD-L1 expression with CD8+ tumor associated lymphocytes was observed in ascites. But in primary and peritoneal tumor tissues PD-1 expression correlated positively with the frequency of CD8+ tumor associated lymphocytes (*r*=0.6, p<0.05) as well as with PD-L1 expression (*r*=0.59, p<0.05; Supplementary Figure S5C).

As Abiko *et al*. showed that IFNγ produced by lymphocytes promotes the expression of PD-L1 [26] we correlated cytokine concentrations with the expression of the gene encoding PD-L1 in solid tumor tissues. Indeed, a positive correlation (*r*=0.62, p<0.05) of IFNγ levels in ascites with the expression of PD-L1 in tumors could be observed. Also serum levels of IFNγ correlated with the expression of PD-L1 in tumors, albeit only as trend and less pronounced (*r*=0.46, p<0.1; Supplementary Figure S5D).

### Immunohistochemical staining for PD-1, PD-L1, and CD8

**Figure 6:**
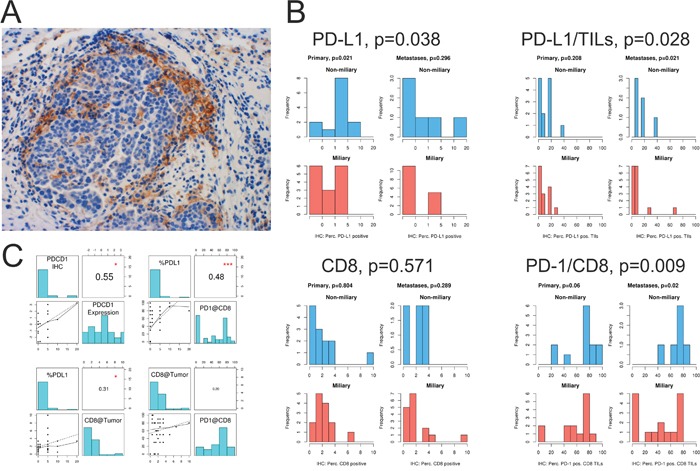
Immune checkpoint analysis of solid tumor tissues **A.** PD-L1 staining of tumor cells at the edge of a tumor nest infiltrated by lymphocytes. **B.** Quantification of IHC staining for PD-L1, PD-1, and CD8. PD-L1, frequency of PD-L1 positive tumor cells; PD-L1/TILs, frequency of PD-L1 positive TILs; CD8, frequency of CD8+ TILs, and PD-1/CD8 frequency of PD-1 positive CD8+ TILs in primary tumors (Primary) and peritoneal implants (Metastases). **C.** Upper panel: correlation of PD-1 (PDCD1) IHC staining and PD-1 (PDCD1) mRNA expression, assessed via RNA-seq. Correlation of PD-L1+ tumor cells and PD-1 positive CD8+ TILs. Lower panel: correlation of PD-L1+ tumor cells with CD8+ TILs, and correlation of CD8+ TILs and PD-1+CD8+ TILS. *p<0.05, ***p<0.001.

To validate the results from RNA sequencing, we stained whole tissue sections of formalin-fixed, paraffin embedded (FFPE) tumor tissues for CD8, PD-1, and PD-L1. In total, 52 tissues (29 primary tumors and 23 peritoneal implants) from 33 patients were analyzed. A representative staining of PD-L1 positive tumor cells infiltrated with immune cells is shown in Figure 6A. PD-L1 staining was assessed in percent of tumor cells and in percent of tumor infiltrating lymphocytes (TILs; Figure 6B). CD8 staining was assessed in percent of intraepithelial TILs and PD-1 staining in percent of CD8+ TILs (Figure 6B).

The frequency of PD-L1 positive tumor cells significantly correlated with PD-L1 gene expression assessed via RNA-seq (*r*=0.55, p<0.05; Figure 6C). Furthermore, significantly more tumor cells and TILs were positive for PD-L1 in non-miliary compared to miliary tissues (p=0.038 and p=0.028, respectively; Figure 6B). Although the frequency of PD-L1 positive tumor cells correlated positively with the observed frequency of CD8+ TILs (*r*=0.31, p<0.05; Figure 6C), no significant differences were observed comparing the amount of CD8+ TILs between non-miliary and miliary tumors (p=0.571, Figure 6B).

PD-1 expressing CD8+ TILs were significantly enriched in non-miliary compared to miliary tumor tissues (p=0.009, Figure 6B). Interestingly, the percentage of PD-L1 positive tumor cells correlated positively with the percentage of PD-1 positive CD8+TILs (*r*=0.48, p<0.001; Figure 6C).

Summarizing, although no evidence for higher infiltration of tumors with CD8+ TILs was observed in non-miliary, more CD8+ TILs showed PD-1 expression than in miliary, indicating a higher T-cell activation in non-miliary. Also, in non-miliary more tumor cells were found to express the ligand PD-L1 which correlated positively with the amount of PD-1 expressing CD8+ TILs.

### Metabolomics

**Figure 7:**
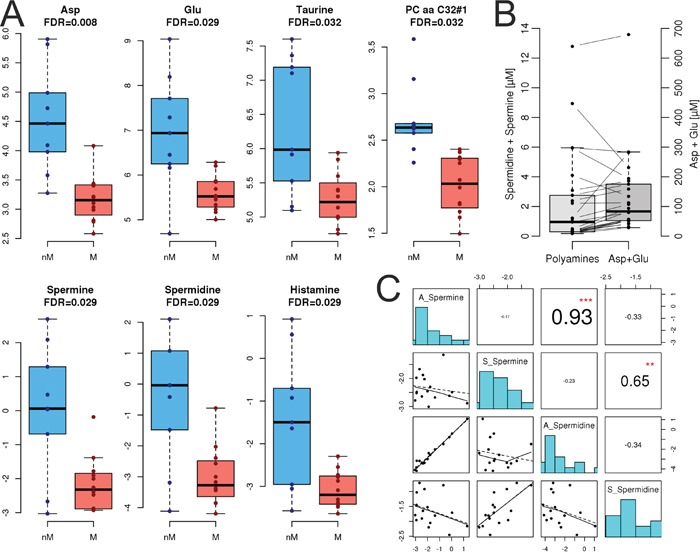
Metabolomics **A.** Quantification of metabolite levels in ascites, separated for non-miliary (nM, blue) and miliary (M, red) tumor spread. Asp, aspartate; Glu, glutamate. The accuracies of histamine were not within the tolerance window of 50–150% in the quality controls and therefore, histamine is not discussed. **B.** Correlation of combined positively charged spermine and spermidine concentrations with combined negatively charged aspartate and glutamate concentrations. **C.** Correlation of spermine and spermidine levels in ascites (A) and serum (S). **p<0.01, ***p<0.001.

To sustain the increased need of energy and building blocks for cell growth and proliferation, tumor cells undergo major metabolic changes. Therefore, we analyzed the levels of 188 metabolites in ascites (nine non-miliary and twelve miliary) and serum (nine non-miliary and 15 miliary) of HGSC patients with respect to tumor spread (Supplementary Table S5). In serum, we found no significant differences between non-miliary and miliary patients in any of the metabolites. In contrast, in ascites the concentrations of six metabolites were significantly lower in miliary patients compared to non-miliary (FDR<0.05); *i.e.* taurine, two non-essential amino acids (aspartate and glutamate), two polyamines (spermidine and spermine), and one unsaturated glycerophospholipid (PC aa C32:1, Figure 7A). Aspartate and glutamate are synthesized independently; the first is member of the oxaloacetate/aspartate family the second of the α-ketoglutarate family. There seems to be a co-occurence of the negatively charged amino acids and the polyamines, albeit the former in a 50-fold excess (Figure 7B). There are transporters, *i.e.* glutamate transporter, which transport glutamate and aspartate from extracellular space into cells, especially in the context of nerve cells. Notably, spermine and spermidine concentrations in ascites and serum correlated very strongly with each other (*r*=0.93 and *r*=0.65, respectively) but not between ascites and serum, where for both small, non-significant negative correlations were revealed (*r*=−0.17 and *r*=−0.23, respectively). Comparing metabolites, significantly associated with spread, two cytokines and chemokines in ascites revealed a strong positive correlation of all these metabolites to IL-16, basic FGF, a weaker positive correlation to MIF (except for glycerophospholipid), and a strong negative correlation to IL-10.

## DISCUSSION

We previously discovered and described the presence of two apparently different modes of tumor spread in the peritoneum of high grade serous ovarian cancer (HGSC) patients and proved differences in overall survival. In our previous publications on the topic we already proposed a microenvironmental impact on peritoneal tumor spread in HGSC patients apart from tumor-intrinsic factors [11, 12].

Indeed, the comprehensive results from the well-defined cohort of HGSC patients proved that patients presenting with non-miliary tumor spread showed more signs of adaptive, tumor directed, immune responses, whereas patients presenting with miliary spread showed more systemic inflammation, indicating a more active innate immune system [27].

Previous flow cytometric analyses in the same patient cohort revealed elevated levels of CD44+CD45-EpCAM- cells in the ascites of patients with non-miliary tumor spread [11]. The lymphocyte homing receptor CD44 was proposed as an ovarian cancer stem cell marker but is also expressed on normal somatic cells (*i.e*. fibroblasts and reactive mesothelial cells) [28]. A higher abundance of shed reactive mesothelial cells in the ascites of non-miliary might reflect an ongoing inflammatory process in the peritoneal cavity of this subtype. To test this hypothesis, we analyzed levels of C-reactive protein (CRP), an opsonin produced in the liver and routinely used as inflammation marker. Levels of CRP were significantly higher in miliary ascites, whereas in blood this association did not reach statistical significance. However, the positive correlation of blood and ascites CRP levels and the association of higher levels of serum CRP with worse prognosis [29] is in line with our previously published results of a worse overall survival of HGSC patients with miliary tumor spread.

As CRP is a rather unspecific marker and does not reflect the types of immune cells present in ascites and tumors we performed in-depth analyses of the immunological and metabolic milieu in blood, ascites, and tumor tissue of these patients. The first step was to compare the molecular subclasses of Tothill *et al*. [7] and the tumor spread patterns in our samples and indeed, we could show that the immunoreactive subtype (C2) is enriched in non-miliary samples, whereas the differentiated subtype (C4) is associated with the miliary subtype, which we previously described to be more epithelial [11]. The immunoreactive subtype (C2) was associated with higher numbers of tumor infiltrating lymphocytes and a better overall survival [9, 10]. IF staining of ascites samples revealed an increase of MO/MA and a decrease of tumor cells in non-miliary compared to miliary. This might result from a better anti-tumor response in non-miliary or from more effective standardized first-line chemotherapy in non-miliary patients since cisplatin and paclitaxel were shown to induce the cytotoxic activity of monocytes against tumor cells, at least in murine models [30–32].

Cytotoxic T-cells (CTL) are major players in tumor surveillance. The enrichment of CD8+ CTL among lymphocytes in non-miliary ascites corresponds to the increased abundance of this population among tumor infiltrating lymphocytes in non-miliary spreading solid tumors. Furthermore, we observed an enrichment of GM-CSF in non-miliary and a positive correlation of this cytokine with the frequency of CD8+ cells in ascites. Among various sources, GM-CSF is also produced by cytotoxic T-cells and associated with anti-tumoral functions exerted by monocytes [19].

A further lymphocyte population, shown to be enriched in non-miliary ascites, was comprised of NKT cells, possibly as response to hypoxic conditions in ascites. The analysis of corresponding RNA-seq data of CD45-enriched immune cells from ascites showed an association of the frequency of NKT cells with the HIF1 signaling pathway. The hypoxia inducible transcription factor HIFα is needed for transcription of angiogenesis related genes and HIF1α mRNA is also upregulated in T-cells upon TCR signaling under hypoxic conditions [33].

The composition of miliary and non-miliary ascites differed also in the prevalence of NK cell subtypes depending on their cytotoxic and cytokine producing abilities [13]. In non-miliary ascites, CD56^bright^ NK cells were predominant whereas in miliary ascites CD56^dim^CD16+ NK cells, known to be highly cytolytically active, were predominant. CD56^bright^ cells were described to be enriched in sites of peripheral inflammation [34], which supports our hypothesis of a tumor-associated adaptive immune response in non-miliary ascites.

Still, various immune cell types were present in miliary ascites and B-cell frequencies were even increased compared to control and non-miliary ascites. Known for their important function as antibody-producing cells and in antigen presentation, B-cells were discussed to display an immune regulatory phenotype in the tumor microenvironment via upregulation of immunosuppressive cytokines [35]. In our samples, IL-10 was significantly upregulated in miliary ascites in comparison to non-miliary and a correlation based network of analyzed cytokines and chemokines proved an association of IL-10 upregulation with MIF downregulation, resulting in lower levels of leukocyte abundance in miliary ascites. Recent studies with B-cell deficient mice showed diminished tumor growth and increased infiltration of tumors with T-cells and NK-cells [36]. Various other studies showed an inhibition of anti-tumor immune responses by B-cells [37, 38]. In ovarian cancer, B-cells were shown to be preferentially enriched in malignant ascites and negatively correlated with the presence of CD8+ T-cells and positively correlated with Tregs [39]. Taken together, the pro-tumoral role of B-cells in inhibiting adaptive immune cell responses to tumor growth might explain the widespread implantation and growth of tumor nodules in patients with miliary tumor spread. The presence of B-cells could facilitate the passage of tumor cells through the ascites by downregulating local immune responses.

Differences in the prevalence of immune cell populations and chemokines in the ascites suggest an important role of the adaptive immune response in tumor spread. Tumor cells of non-miliary and miliary spreading tumors might not elicit the same immune responses. Since miliary tumors were shown to be more epithelial [11] and associated with the differentiated molecular subtype (C4), surface (tumor-) antigens might not stimulate an effective response of the adaptive immune system compared to the more mesenchymal type of non-miliary spreading tumors [11]. Still, the increase in CRP and the elevated levels of cytotoxic NK (NK1) cells in miliary patients suggest, inflammatory processes, at least innate ones [27], are ongoing in the peritoneal cavity. In miliary, a shift of the immune system from tumor surveillance to pro-tumorigenic actions seems possible, supported by the increased presence of B-cells and elevated IL-10 concentrations in the ascites.

Non-miliary tumors on the other hand are characterized by anti-tumoral immune responses of the adaptive immune system. In concordance with the increased presence of CD8+ T-cells in non-miliary ascites, non-miliary tumors showed higher levels of CD8+ tumor associated lymphocytes in FACS analysis and more PD-1+CD8+TILs in IHC. Nevertheless, these tumors also employed strategies of immune evasion. Checkpoint inhibitors like PD-1 and CTLA-4 augment immune reactions and tumors cells were shown to hijack these function in order to evade the immune system by downregulation of T-cell mediated immune responses [6]. In RNA-seq analysis, we found elevated levels of PD-L1 mRNA in ascites tumor cells and tumor tissues in non-miliary compared to miliary. Furthermore, PD-1 mRNA levels (expressed by TILs) were upregulated in non-miliary CD45 positive tumor associated immune cells. These results could be validated by IHC of whole tissue sections from primary tumors and peritoneal implants. Despite the immune regulatory role of the ligand PD-L1, high PD-1 and PD-L1 expression in high grade serous ovarian cancer was associated with a positive prognosis [40], explainable with the positive correlation of PD-1 and PD-L1 expression with the presence of TILs, as observed in non-miliary tumors. The additional insight that tumor cells showed upregulation of PD-L1 preferentially in regions with immune cell infiltration may be explained by a study by Abiko *et al.* where they could show that IFNγ produced by lymphocytes promotes PD-L1 expression [26]. Supporting this finding, we observed a positive correlation of IFNγ levels in ascites and PD-L1 expression of tumors.

Furthermore, polyamines (*e.g.* spermine and spermidine) were recently discussed to act as immunomodulators and blockade of polyamines was shown to promote anti-tumor immunity [41]. Analysis of metabolic differences between miliary and non-miliary subtypes of HGSC showed an increased abundance of the polyamines spermine and its progenitor spermidine in non-miliary ascites. Although spermine was shown to inhibit pro-inflammatory cytokine synthesis [42], from our data polyamine levels correlated negatively with the anti-inflammatory cytokine IL-10 but positively with IL-16, basic FGF, and MIF. IL-16 is a pro-inflammatory and pro-angiogenic cytokine which is produced by lymphocytes and some epithelial cells, probably also by tumor cells, and attracts CD4+ cells, especially T-cells. It was shown that IL-16 expression and serum abundance increases from healthy to benign tumors to early and ultimately to late stages of ovarian cancer and is positively correlated with tumor-associated microvessels [43].

The results from targeted metabolomics could also be explained very speculatively by an increased neo-neurogenesis [44] of non-miliary tumors. Three of the six metabolites, significantly elevated in non-miliary, are neurotransmitters (aspartate, glutamate, and taurine) and extracellular polyamines are known to be necessary for adult neurogenesis, at least in rodents [45]. Furthermore, accumulation of IL-16+ microglia was shown in human fetal brains in zones of neuronal proliferation, migration, and differentiation [46].

In conclusion, non-miliary tumors seem to be more immunogenic and show signs of an elevated adaptive immune response. Albeit better prognosis, non-miliary tumors also show active immune evasion, *e.g.* evidenced by tumor cells expressing PD-L1 that might contribute to disease progression and metastasis. On the other hand, miliary tumors seem to provoke innate immune responses and show signs for local and systemic inflammation (*i.e.* CRP). The tumor-directed adaptive immune response in miliary seems to be counteracted by the induction of immune repressing factors, possibly associated to B-cells and Tregs.

With the growing field of immunotherapy and several agents already in clinical trials for therapy of HGSC, the characteristics of the group of non-miliary tumors renders these tumors as accessible for checkpoint inhibitors like Ipilimumab, Pembrolizumab, or Avelumab. Patients with miliary tumors on the other hand might benefit from therapies boosting the immune system while targeting especially epithelial cells, like Catumaxomab. Still, the impact of the comprehensive results from this study, showing differences in the immune reactions associated to differences in peritoneal tumor spread, on possible treatment options needs to be elucidated in further studies.

## MATERIALS AND METHODS

### Study design

Tumor tissues from ovarian and peritoneal tumors, peripheral blood, and ascites of chemotherapy naïve HGSOC patients were collected consecutively at the Medical University of Vienna from February 2012 to July 2014. Furthermore, ascites and blood of healthy controls were included. Approval for this study was obtained by the ethical review board (nos. 366/2003 and 793/2011) and all patients signed an informed consent. A pathologist specialized for gynecological malignancies assessed the clinicopathological characteristics as histology, FIGO stage, and grade and supervised the immunohistochemistry stainings and analysis.

### Patients material

Blood was collected in EDTA-tubes before surgery. To get rid of red blood cells, erythrocytes were lysed in ammonium chloride lysis buffer and the remaining cells were cryopreserved in liquid nitrogen with 5% DMSO in plasma. Serum was obtained from serum tubes by centrifugation and stored at −80°C. Ascites cells were pelleted by centrifugation and cryopreserved in liquid nitrogen with 5% DMSO in cell free ascites supernatant for FACS analysis. Additionally, aliquots of cell free ascites supernatant were stored at −80°C for subsequent immunoassay analysis. For gene expression analysis, immune cells were enriched by isolation of CD45+ cells from filtered ascites, using Dynabeads (Invitrogen, Carlsbad, CA, USA) and a magnetic bead-based cell separation device. Tissue slices from ovarian and peritoneal tumors were obtained during surgery. The samples were minced, digested with 1.04 U ml^−1^ Liberase DH research grade (Roche, Basel, Switzerland) while stirring at 37°C for one hour and the resulting cell suspension was enriched for immune cells as mentioned above. Furthermore, tissue cell suspensions, depleted of EpCAM+ tumor cells with the same method, were cryopreserved in liquid nitrogen for subsequent FACS analysis. Tumor cells from the same samples were enriched as described before [11].

### Flow cytometric analysis (FACS)

Surface expression of immune cell markers was measured by direct immunofluorescence using combinations of the following fluorophore-conjugated mouse anti-human antibodies, obtained from BD Bioscience (NJ, USA). First panel: V500 anti-CD45 (HI30), APC-H7 anti-CD3 (clone SK7), PerCP anti-CD8 (clone SK1), FITC anti-CD4 (clone RPA-T4), APC anti-CD25 (clone M-A251), PE anti-CD127 (clone HIL-7R-M21). Second panel: V500 anti-CD45, APC-H7 anti-CD3, APC anti-CD19, PerCP-Cy5.5 anti-CD27 (clone M-T271), PE anti-CD56 (clone NCAM16.2), FITC anti-CD16 (clone B73.1), PE-Cy7 anti-HLA-DR (clone G46-6). After thawing, all samples were stained for dead cells using the LIVE/DEAD® Fixable Dead Cell Stain (Invitrogen^TM^, Carlsbad, CA, USA) according to the manufacturer's instructions. Afterwards, incubation with the antibody mix was done for 30 minutes, followed by acquisition on a BD FACSVerse flow cytometer, equipped with three lasers (405 nm, 488 nm, and 633 nm). Analysis was performed using FlowJo software (v7.6.2, Tree Star, Inc., Ashland, OR, USA). Cell doublets and dead cells were excluded from analysis and different cell populations were gated according to expression of surface markers.

### RNA preparation

RNA was prepared from previously frozen samples stored in QIAZOL lysis buffer with the miRNeasy® Mini Kit (Qiagen, Hilden, Germany) according to manufacturer's instructions for preparation of RNAs longer than 200 nucleotides. The quality was assessed by analysis on an Agilent 2100 Bioanalyzer (Santa Clara, CA, USA) and samples with RNA Integrity Numbers (RIN) >7 were used for library preparation.

### Library preparation and RNA-Seq

Sequencing libraries were prepared from 200 ng total RNA >200 nt, quantified with NanoDrop ND-1000 (Thermo Fisher Scientific, Waltham, USA) spectrometer and RiboGreen RNA Reagent (Invitrogen, Carlsbad, CA, USA). NEBNext Poly(A) mRNA Magnetic Isolation Module (NEB: New England Biolabs, Ipswich, MA, USA) was used for poly(A) positive mRNA selection and Illumina compatible libraries were prepared with NEBNext® Ultra™ Directional RNA Library Prep Kit for Illumina (NEB) with halved reaction volumes according to the manufacturer's instructions. After quality assessment on a DNA High Sensitivity chip (Bioanalyzer 2100), libraries were quantified with digital PCR (QX100™ Droplet Digital™ PCR System, BioRad, Hercules, CA, USA) using the ddPCR™ Library Quantification Kit for Illumina (BioRad). For each lane of the Illumina HiSeq 2000 system (San Diego, CA, USA) eight libraries were pooled equimolarily and sequenced for 50 bp paired ends.

### Multiplexed immunoassays

Chemokines and growth factors in blood and ascites were assessed using the commercially available kits for multiplexed immunoassay Bio-Plex Pro^TM^ Human Chemokine Panel, 40-plex and Human Cancer Biomarker Panel 1, 16-plex (Bio-Rad Laboratories, Hercules, CA, USA). Ascites supernatant and serum were diluted 1:6 after thawing and immunoassays were prepared according to manufacturer's instructions and analyzed on a Bio-Plex 200® system (Bio-Rad). The co-occurrence network was reconstructed from ascites cyto/chemokine concentration values using Gaussian graphical models (R-package GGMselect v0.1-10 (http://fr.arxiv.org/abs/0907.0619). Isomap (a non-linear dimensionality reduction approach) was performed with R-package RDRToolbox v1.20.0 [47] using cyto/chemokines significantly associated with spread.

### Laboratory parameters

Levels of C-reactive protein (CRP), albumin (ALB), triglycerides (TRIG), cholesterol (CHOL), low-density lipoprotein (LDL), and high-density lipoprotein (HDL) were assessed in serum and cell free ascites supernatants. Frozen samples (−80°C) were thawed, diluted with 1x PBS (1:5 for plasma, 1:2 for ascites supernatants) and analyzed on a cobas 8000 modular analyzer system (Hoffmann-LaRoche, Basel, Switzerland) using the respective tests, distributed by Roche diagnostics (CRPL3, ALBT2, TRIGL, CHOL2, LDL_C, and HDLC3) in an ISO9001:2008 certified laboratory (Department of Laboratory Medicine, Medical University of Vienna).

### Metabolomics

Targeted metabolomics on cell-free ascites and serum of a subset of 19 HGSC patients was performed using AbsoluteIDQ p180 kits (Biocrates Life Sciences AG, Innsbruck, Austria). The kit allows the identification and (semi-) quantification of metabolites by LC- and flow injection analysis (FIA)-MRM. The samples were analyzed on an AB SCIEX QTrap 4000 mass spectrometer (Framingham, MA, USA) using an Agilent 1200 RR HPLC system (Agilent Technologies, Santa Clara, CA, USA), which were operated with Analyst 1.6.2 (AB SCIEX). The chromatographic column was obtained from Biocrates. The serum samples and additional blanks, calibration standards and quality controls were prepared according to the user manual. All amino acids and biogenic amines were derivatized with phenylisothiocyanate. The experiments were validated using the supplied software (MetIDQ, Version 5-4-8-DB100-Boron-2607, Biocrates). Histamine, methioninesulfoxide, symmetric dimethylarginine, α-aminoadipic acid and trans-4-hydroxyproline did not pass quality controls and therefore, were not considered for data interpretation.

### Immunostaining

Immunohistochemistry (IHC) was performed on formalin-fixed, paraffin-embedded (FFPE) whole tissue sections of ovarian tumors and peritoneal implants. The IHC procedure was performed on an automated LEICA BOND III Immunhistostainer (Leica Biosystems, Wetzlar, Germany) with the following parameters. Heat induced epitope retrieval for 20 minutes using BOND Epitope Retrieval Solution, BOND Polymere Refine Detection Kit DS9800 with the protocol for primary antibodies for 30 minutes. Antibodies used were DAKO CD8 M7103 monoclonal mouse anti human antibody, diluted 1:100 in BOND Primary Antibody Diluent, Cell Marque 315M-96 monoclonal mouse anti human antibody PD1 (1:50), and Cell Signaling 13684 monoclonal rabbit anti human antibody (1:100) PD-L1. Kidney sections were used as positive control. Examination of samples was done by two independent observers including a pathologist. PD-L1 expression was determined using a scoring system based on staining intensity (0-3) and percentage of tumor cells positively stained.

Multicolor IF staining of EpCAM, CD45, CD14, and CD16 was performed according to standard protocols. Shortly, sections of agarose and paraffin embedded ascites blocks were deparaffinized, rehydrated, and subsequent heat induced epitope retrieval was performed using EDTA pH 8. Blocking was done with Ultra V Block (Thermo Fisher Scientific, MA, USA) and slides were incubated with primary antibodies for 60 min. Following primary antibodies were used: anti-CD45 (dilution 1:1,000, source rat, isotype IgG2b, clone orb96558, Biorbyt, Cambridge, UK), anti-CD14 (dilution 1:250, source rabbit, isotype IgG, clone EPR3653, Novus Biologicals, CO, USA), anti-CD16 (dilution 1:50, source mouse, isotype IgG2a, clone 2H7, Thermo Scientific), and anti-EpCAM (dilution 1:300, source mouse, isotype IgG1, clone VU1D9, Cell Signaling, Cambridge, UK). After washing, secondary antibodies (goat anti-mouse IgG2a Alexa Fluor® 488, goat anti-rat Alexa Fluor® 555, goat anti-mouse IgG1 Alexa Fluor® 647, goat anti-rabbit Alexa Fluor® 750, all from Life Technologies, CA, USA) were applied and after 60 min incubation, nuclei were counterstained with DAPI. Slides were scanned with a TissueFAXS fluorescence microscope (TissueGnostics, Vienna, Austria) and quantification of cells was performed using the cell analyzing software CellProfiler v.2.1.1.

### Bioinformatic and statistical analyses

Bioinformatic and statistics was done in R version 3.2.1 (Vienna, Austria). Reads from RNA-sequencing were demultiplexed, mapped to the human genome (HG19), counted into the gene model from Gencode (version 19), normalized, and filtered as described elsewhere [11]. Differential gene expression was analyzed using the R-package limma v3.26.3 [48] and pathway analysis was performed with signaling pathway impact analysis (R-package SPIA v2.22.0, [49]) as described before [11]. Samples were stratified according to the subgroups proposed by Tothill *et al.* [7], the TCGA [8], and in angiogenic and non-angiogenic [24] using the R-package genefu v2.2.0 (https://www.pmgenomics.ca/bhklab/software/genefu). These subgroups were compared to the proposed tumor spread types miliary and non-miliary corrected for tumor tissue origin (P, M, A, and S) and patient. In all statistical analyses two-sided p-values below 0.05 or false discovery rates (FDRs) below 0.2 were considered as statistically significant. Correction for multiple testing according Benjamini-Hochberg was performed, if necessary. Associations between variables were assessed using Spearman's rank correlation.

## SUPPLEMENTARY DATA












