# Supplementary material for: Role of the immune system in the peritoneal tumor spread of high grade serous ovarian cancer
Source: Oncotarget. 2016 Aug 3;7(38):61336–54. doi: 10.18632/oncotarget.11038 (PMC5308655; doi:10.18632/oncotarget.11038)
Supplement: Supplementary file 1 [file oncotarget-07-61336-s001.pdf]

Role of the immune system in the peritoneal tumor spread of high grade serous ovarian cancer

SUPPLEMENTARY DATA

REFERENCES

1. Auer K, Bachmayr-Heyda A, Aust S, Sukhbaatar N, Reiner AT, Grimm C, Horvat R, Zeillinger R, Pils D. Peritoneal tumor spread in serous ovarian cancer-epithelial mesenchymal status and outcome. Oncotarget. 2015; 6:17261-17275. doi: 10.18632/oncotarget.3746.

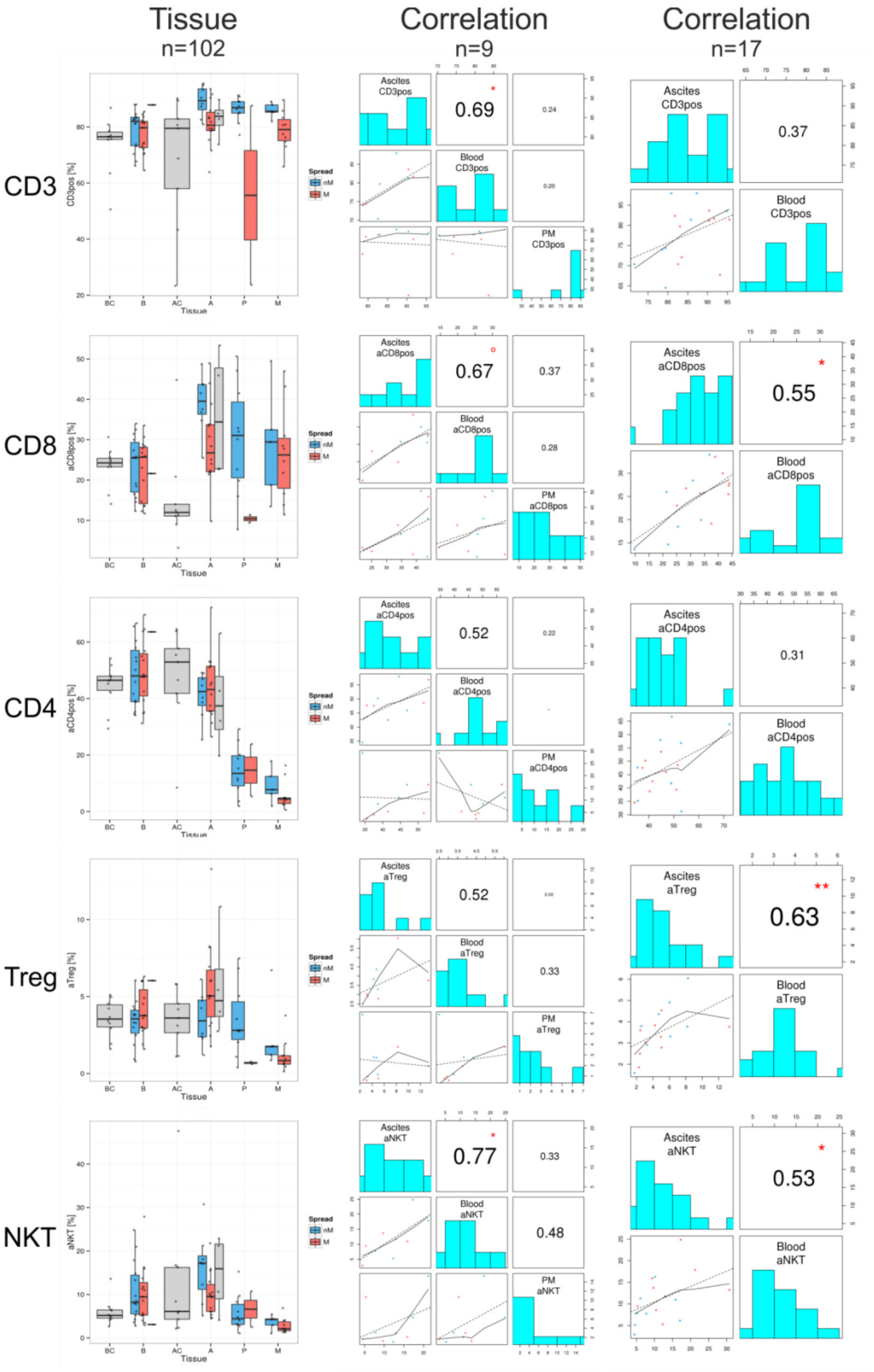
Tissue
n=102
Correlation
n=9
Correlation
n=17
CD3
CD8
CD4
Treg
NKT
Spread
nM
M
Tissue
Ascites CD3pos
Blood CD3pos
PM CD3pos
0.69
0.24
0.20
0.37
Ascites aCD8pos
Blood aCD8pos
PM aCD8pos
0.67
0.37
0.28
0.55
Ascites aCD4pos
Blood aCD4pos
PM aCD4pos
0.52
0.22
0.31
Ascites aTreg
Blood aTreg
PM aTreg
0.52
0.33
0.63
Ascites aNKT
Blood aNKT
PM aNKT
0.77
0.33
0.48
0.53

(*Continued*)

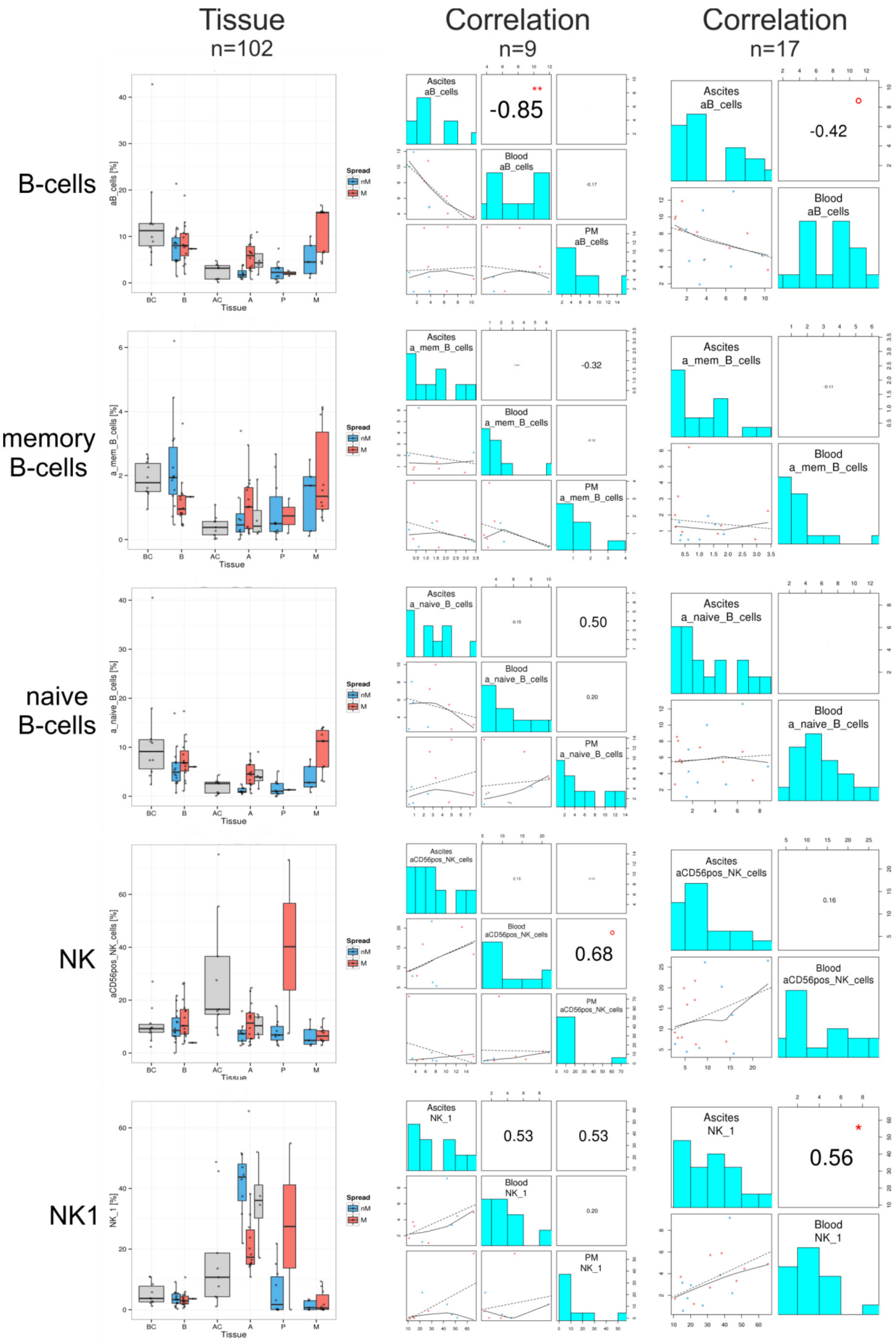
Tissue
n=102
Correlation
n=9
Correlation
n=17
B-cells
memory
B-cells
naive
B-cells
NK
NK1
Spread
nM
M
Tissue
Ascites
aB_cells
Blood
aB_cells
PM
aB_cells
-0.85
-0.42
Ascites
a_mem_B_cells
Blood
a_mem_B_cells
PM
a_mem_B_cells
-0.32
Ascites
a_naive_B_cells
Blood
a_naive_B_cells
PM
a_naive_B_cells
0.50
Ascites
aCD56pos_NK_cells
Blood
aCD56pos_NK_cells
PM
aCD56pos_NK_cells
0.68
0.16
Ascites
NK_1
Blood
NK_1
PM
NK_1
0.53
0.53
0.56

(Continued)

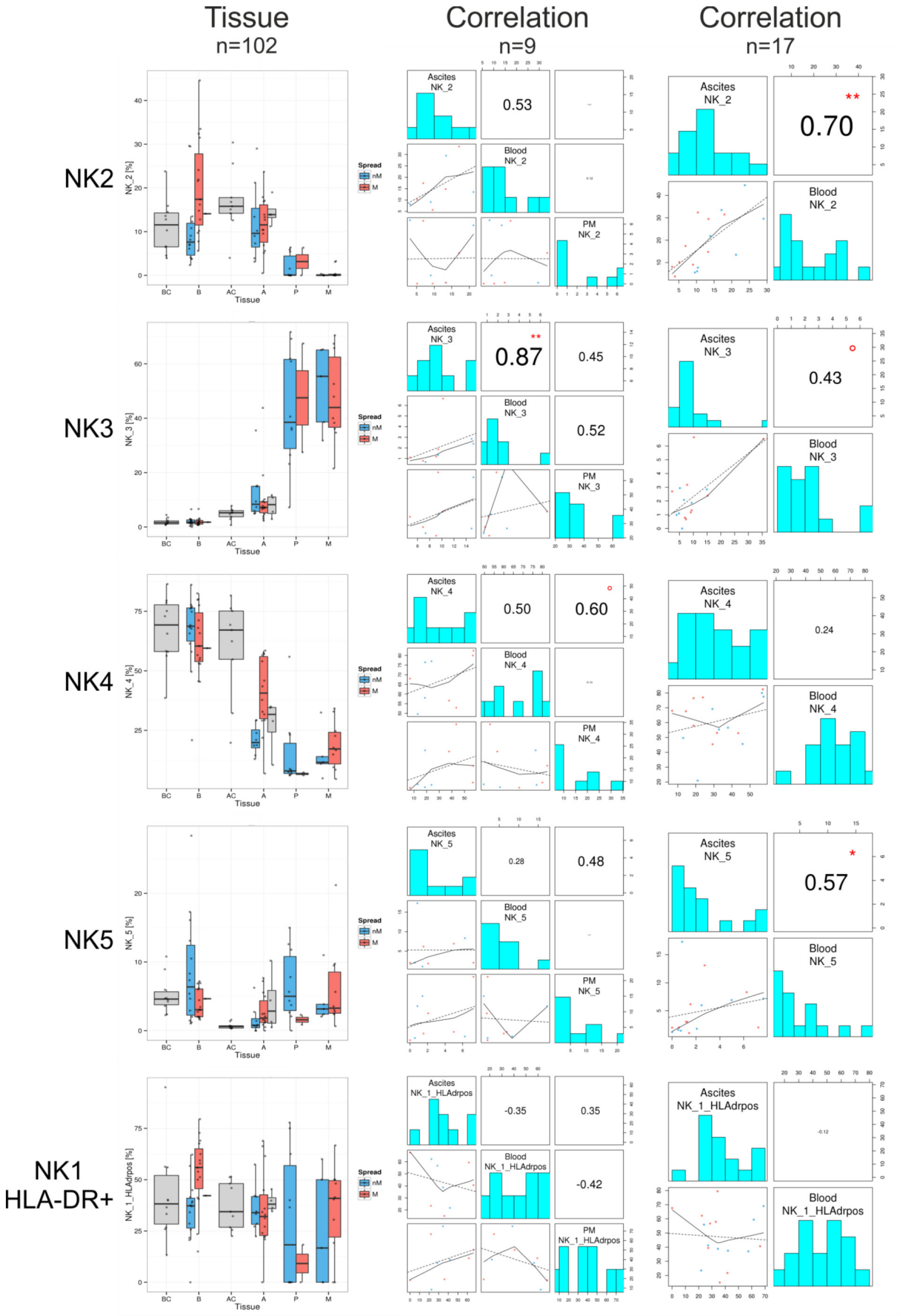

Tissue
n=102
Correlation
n=9
Correlation
n=17
NK2
NK3
NK4
NK5
NK1
HLA-DR+
Spread
nM
M
Tissue
BC
B
AC
A
P
M
Ascites
NK_2
Blood
NK_2
PM
NK_2
0.53
0.70
Ascites
NK_3
Blood
NK_3
PM
NK_3
0.87
0.45
0.52
0.43
Ascites
NK_4
Blood
NK_4
PM
NK_4
0.50
0.60
0.24
Ascites
NK_5
Blood
NK_5
PM
NK_5
0.28
0.48
0.57
Ascites
NK_1_HLAdrpos
Blood
NK_1_HLAdrpos
PM
NK_1_HLAdrpos
-0.35
0.35
-0.42
-0.12

(Continued)

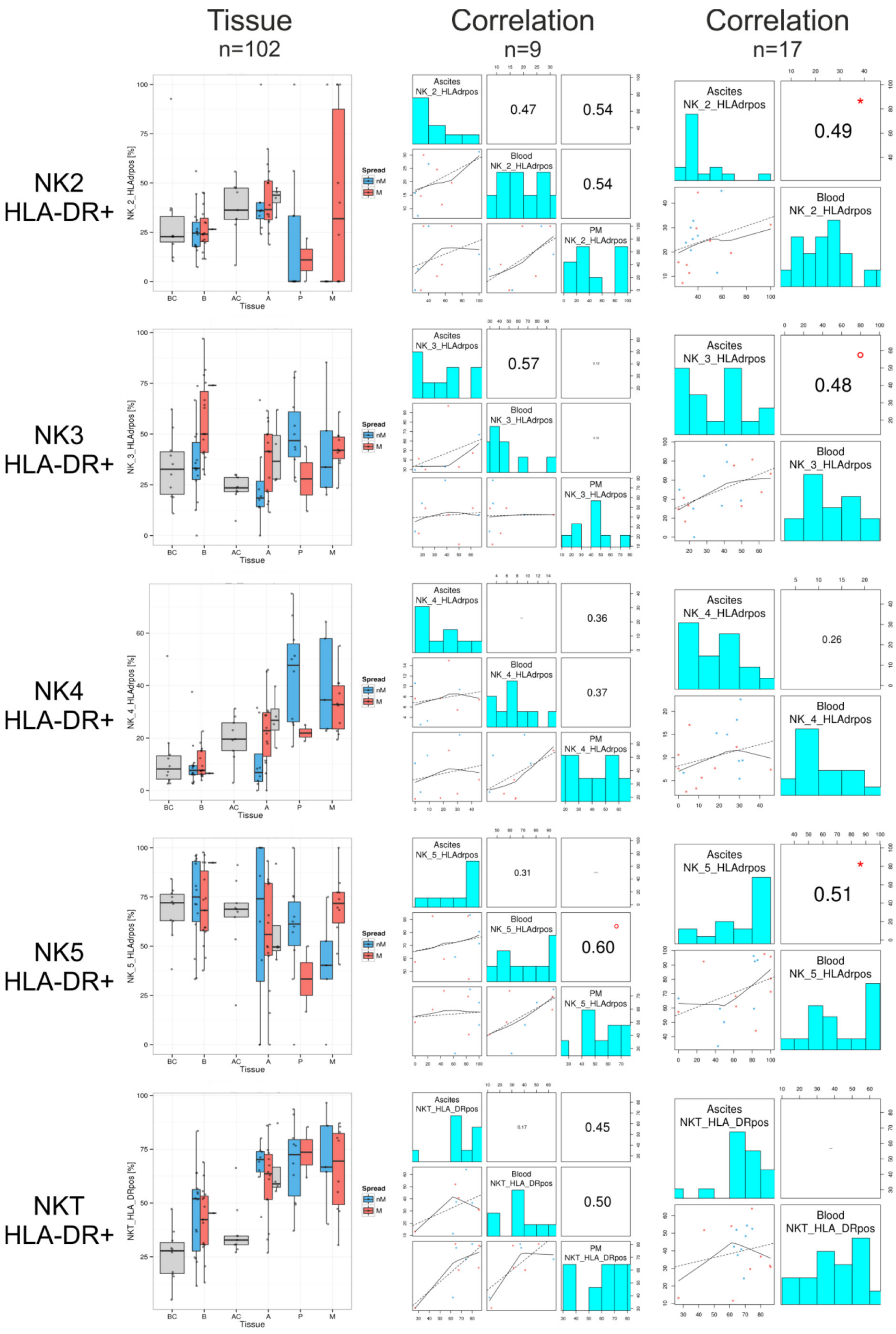

Supplementary Figure S1: Lymphocyte populations in blood, ascites, and solid tumors. From left to right: boxplots representing the calculated frequencies of the respective cell populations in percent of lymphocytes. BC, control blood; B, blood from HGSC patients; AC, control ascites; A, malignant ascites; P, primary tumor; M, peritoneal implant (metastasis); grey bars, blood/ascites with indeterminable spread type. Spearman's correlations of the respective cell populations in ascites, blood, and tumor tissues. Size coded values in the top-right square represent correlation coefficients and significance levels are indicated by asterisks (°p<0.1, *p<0.05, **p<0.01, ***p<0.001, Spearman's correlations). Histograms of corresponding values are shown in diagonal boxes in cyan. Solid and dashed lines in the bottom-left square represent fitted LOESS and linear regressions, respectively.

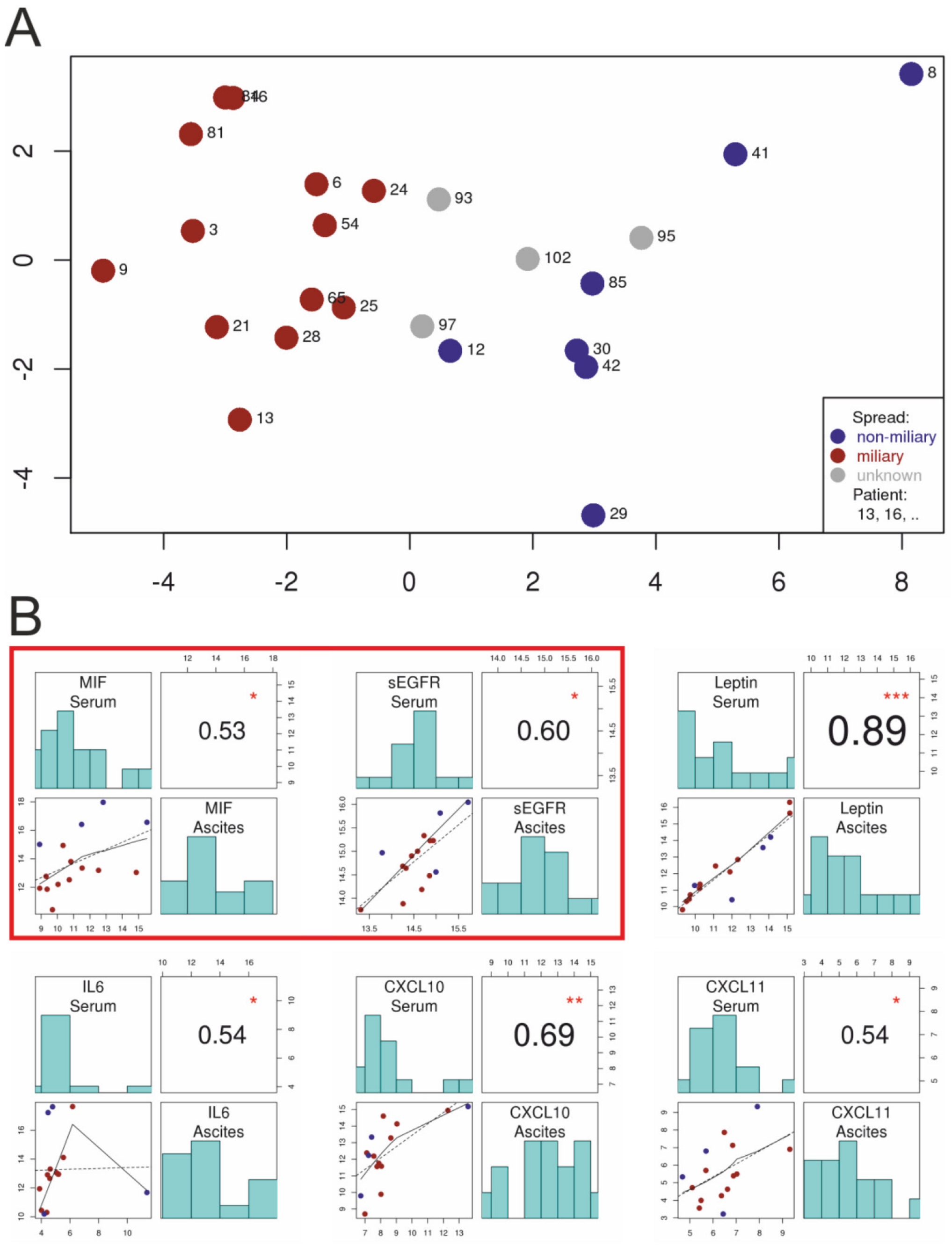

Supplementary Figure S2: Cyto/chemokines associated with spread type and correlations of cyto/chemokine concentrations in ascites and serum. A. Isomap, constructed from chemokines and cytokines differentiating significantly between miliary and non-miliary (n=20). Each dot represents one patient. Blue, non-miliary; red, miliary; grey, tumor spread not determined. **B.** Significantly correlated chemokines and cytokines in ascites and serum, *p<0.05, **p<0.01, ***p<0.001. Red frame: chemokines with statistically significant differences between miliary and non-miliary. Description of plots as in Supplementary Figure S1.

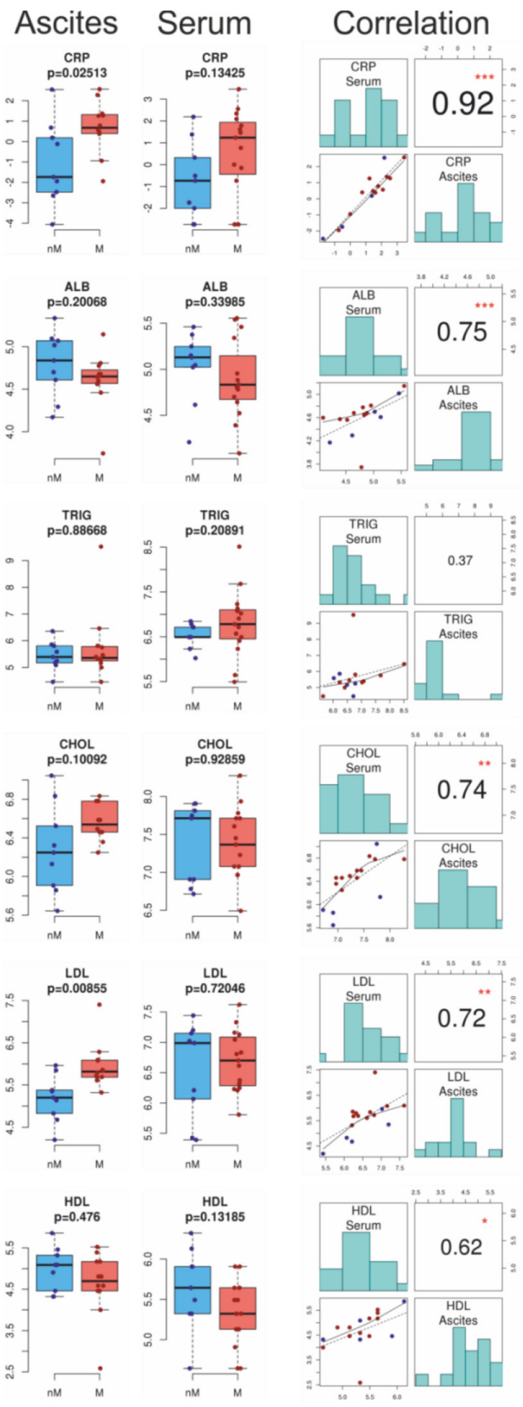

Supplementary Figure S3: Laboratory parameters and correlations between ascites and serum. Calculated values of different laboratory parameters in ascites and serum of HGSC patients, separated in non-miliary (nM, blue) and miliary (M, red). Right: correlations of laboratory parameter in ascites and serum. *p<0.05, **p<0.01, ***p<0.001. CRP, C-reactive protein; ALB, albumin; TRIG, triglycerides; CHOL, cholesterol; LDL, low-density lipoprotein; and HDL, high density lipoprotein. Description of plots as in Supplementary Figure. S1 (colors represent samples from non-miliary (blue) or miliary (red) samples).

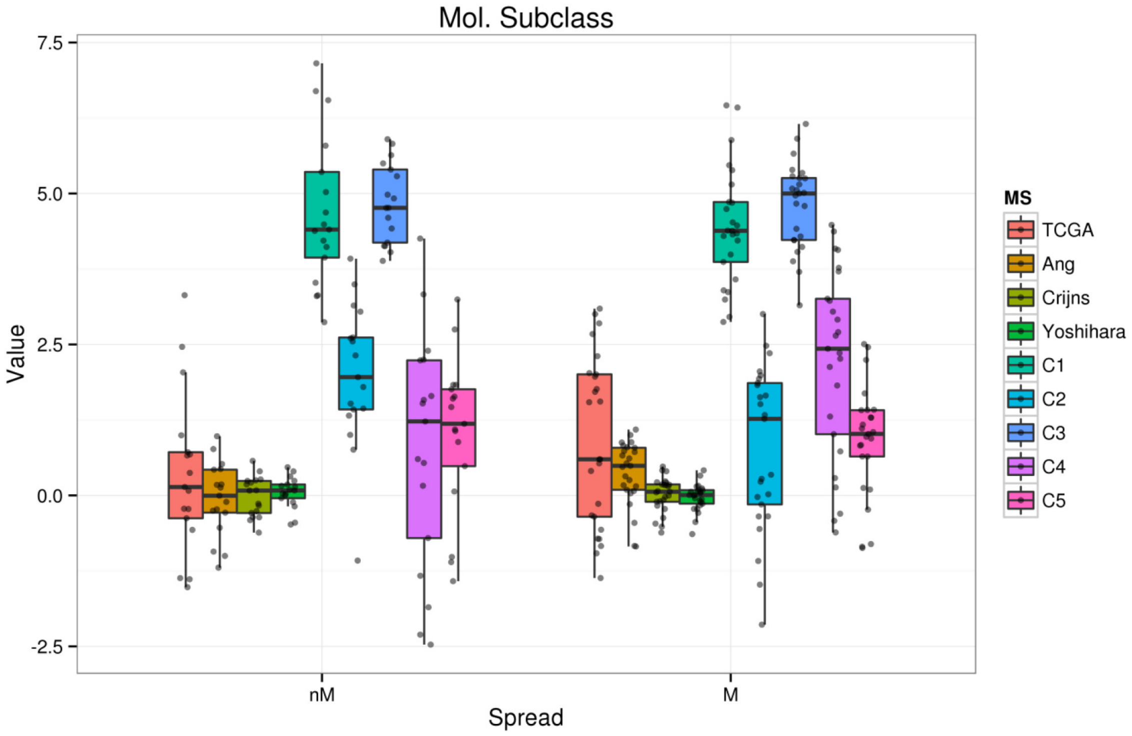

Supplementary Figure S4: Molecular subclasses and tumor spread. Classification of non-miliary and miliary tumor cells from the ascites according to the TCGA subtypes (high, "high risk" with worse overall survival), Angiogenesis (low, more angiogenesis), and molecular subtypes (C1-C5).

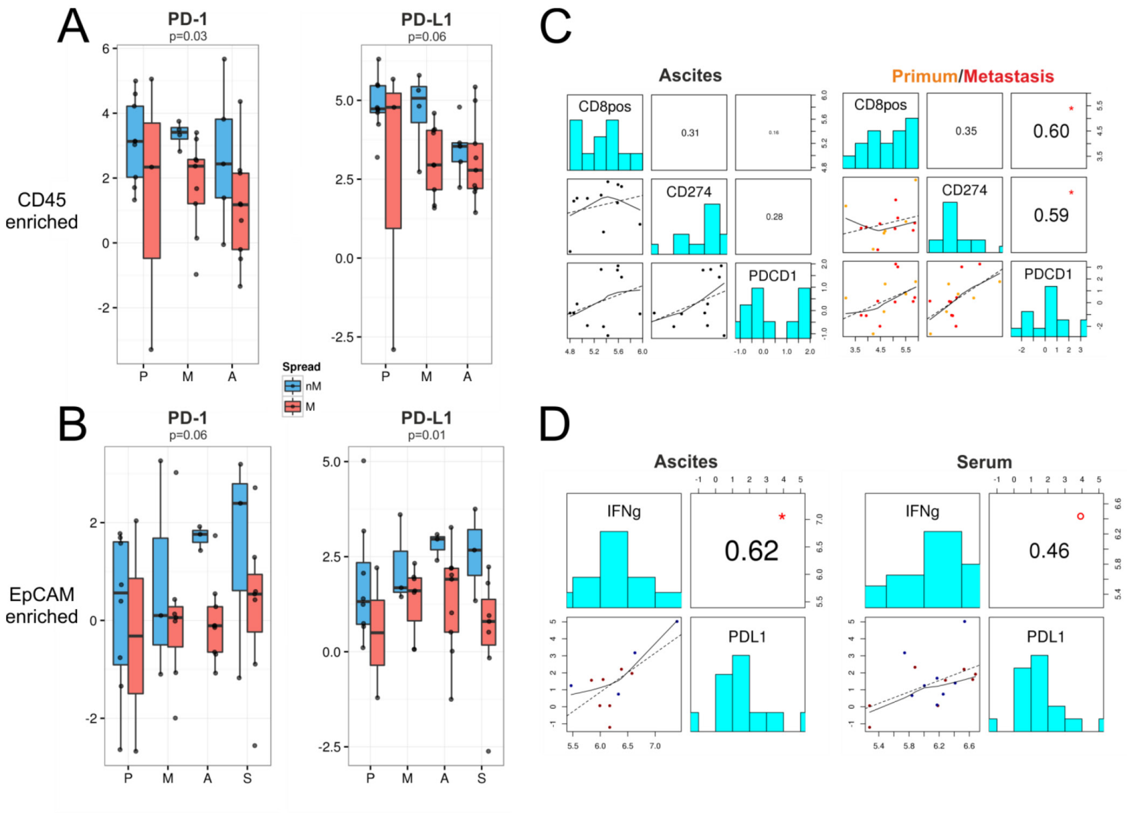

Supplementary Figure S5: PD-1 and PD-L1 expression in tumor and immune cells. A. Boxplots representing the relative gene expression values for the genes encoding PD-1 and PD-L1, respectively, in primary tumors (P), peritoneal implants (M, metastasis), and ascites (A), separated for non-miliary (nM, blue) and miliary (M, red). Shown are data from RNA-seq of CD45-enriched samples. **B.** Boxplots representing the relative gene expression values for the genes encoding PD-1 and PD-L1, respectively, in the same tissues as described above and in spheroids from ascites (S). Shown are data from RNA-seq of EpCAM-enriched samples [1]. **C.** Correlation of the frequency of CD8+ T-cells (FACS) and the expression of PD-L1 (CD274) and PD-1 (PDCD1) in ascites (left) and tumor tissues (right, assess with RNA-seq). **D.** Correlation of INFγ levels with PD-L1 expresssion in ascites (left) and serum (right, RNA-seq). Description of plots as in Supplementary Figure S1 (colors represent samples from non-miliary (blue) or miliary (red) samples).

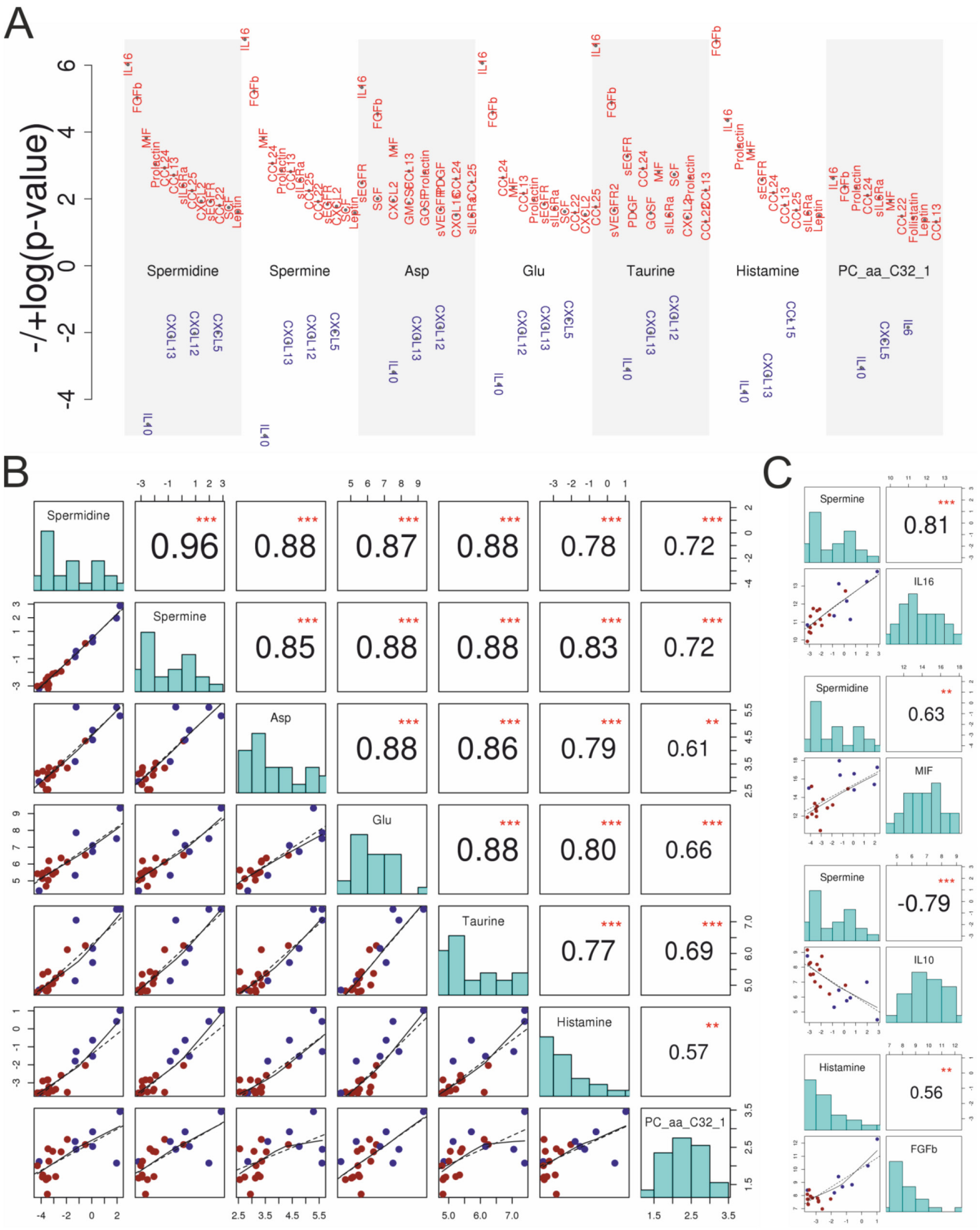

Supplementary Figure S6: Targeted metabolomics and correlation with cyto/chemokines. A. Positive (red) and negative (blue) correlations of chemokines and cytokines with metabolites in ascites; y-axis, -(negative correlation) or +(positive correlation) log(p-value) **B.** Correlation plots of all significantly differentially abundant metabolites in ascites of non-miliary and miliary patients. **C.** Correlation plots of metabolites with chemokines and cytokines in ascites, Description of plots as in Supplementary Figure S1 (colors represent samples from non-miliary (blue) or miliary (red) samples).

Supplementary Table S1: Frequencies of cell populations in blood (B), control blood (BC), ascites (A), control ascites (AC), and primary tumors (P) and peritoneal implants (M) of HGSC patients as assessed with flow cytometry.

See Supplementary File 1

Supplementary Table S2: Results from multiplexed immunoassay. logFC, log fold change of chemo/cytokine concentrations between miliary and non-miliary samples. + up in miliary, - down in miliary; adj. P.val, false discovery rate (FDR).

See Supplementary File 2

Supplementary Table S3: Results from RNA-seq analysis and comparisons of gene expressions between miliary and non-miliary in total (MvsNM), only in ascites (A_MvsNM), and only in solid tumor tissues (PM_MvsNM).

See Supplementary File 3

Supplementary Table S4: Correlation of miliary and non-miliary with different subgroups. *p<0.05, **p<0.01, ***p<0.001.

See Supplementary File 4

Supplementary Table S5: Results from metabolomics. logFC, log fold change of metabolite concentrations between miliary and non-miliary ascites. + up in miliary, - down in miliary; adj. P.val, false discovery rate (FDR).

See Supplementary File 5
